# Triglyceride levels and all-cause mortality in patients with left main coronary artery disease undergoing percutaneous coronary intervention

**DOI:** 10.3389/fcvm.2026.1751611

**Published:** 2026-04-23

**Authors:** HongJing Lu, Yufeng Yan, Haimei Xu, Yingying Zhao, Song Lin, Yaguo Zheng

**Affiliations:** Department of Cardiology, Nanjing First Hospital, Nanjing Medical University, Nanjing, Jiangsu, China

**Keywords:** LM coronaryartery disease, long-term mortality, percutaneous coronary intervention, prognosis, triglyceride

## Abstract

**Backgrounds:**

Triglycerides (TG) and triglyceride-rich lipoproteins contribute to the development and progression of atherosclerosis. However, the prognostic value of TG levels in patients with left main coronary artery disease (LMCAD) remains unexplored. This study aimed to examine the association between TG levels and long-term mortality within this population.

**Methods:**

We conducted a single-center retrospective study of 2,778 patients with LMCAD undergoing percutaneous coronary intervention (PCI). We modeled the association between TG levels and the hazard ratio (HR) for mortality using restricted cubic splines (RCS). An optimal TG cutoff for stratification was identified using the maximum selected rank statistic, and patients were then divided into two groups. We assessed the proportional hazards assumption with plots of Schoenfeld residuals. The primary endpoint was all-cause death. Secondary endpoints included cardiovascular death, myocardial infarction, stroke, stent thrombosis, and target vessel revascularization.

**Results:**

Over a mean follow-up of 47.4 ± 30.3 months, 351 (12.6%) patients died, including 207 cardiovascular deaths. Restricted cubic spline analysis showed a linear inverse relationship between TG levels and all-cause mortality. An optimal TG cutoff of 0.93 mmol/L was identified, dividing patients into high-TG (*n* = 2214) and low-TG (*n* = 564) groups. The low-TG group had significantly higher all-cause mortality (17.2% vs. 11.5%, log-rank P<0.001). After multivariable adjustment, low TG remained independently associated with a higher risk of all-cause mortality [adjusted HR: 1.379; 95% confidence interval (CI): 1.069–1.778; *P* = 0.013]. The low-TG group was also associated with a higher risk of cardiovascular mortality (9.4% vs. 7.0%, log-rank *P* = 0.011). No significant associations were observed between TG levels and other secondary endpoints. Subgroup analyses confirmed the consistent prognostic value of TG across clinical subgroups.

**Conclusion:**

Our findings indicate that low TG levels are an independent prognostic factor for all-cause mortality among LMCAD patients undergoing PCI.

## 1 Introduction

The left main (LM) coronary artery supplies 60%–75% of the left ventricular myocardium. Consequently, LM lesions pose a high risk and are associated with poor outcomes. Although coronary artery bypass grafting (CABG) remains the gold standard, advances in next-generation drug-eluting stents and intravascular imaging have established percutaneous coronary intervention (PCI) as a robust alternative (1). Hypertriglyceridemia is a known risk factor for pancreatitis and atherosclerotic cardiovascular disease (ASCVD) (2, 3). Current evidence suggests that while triglycerides (TG) themselves may not be directly atherogenic, the cholesterol content within TG-rich lipoproteins (termed remnant cholesterol) contributes significantly to residual cardiovascular risk (4–6). However, the prognostic value of serum TG levels in LM lesions remains poorly defined. Therefore, this study aimed to investigate the association between baseline serum TG levels and long-term mortality in patients undergoing PCI for significant left main coronary artery disease (LMCAD).

## 2 Method

### 2.1 Study population

**Figure 1:**
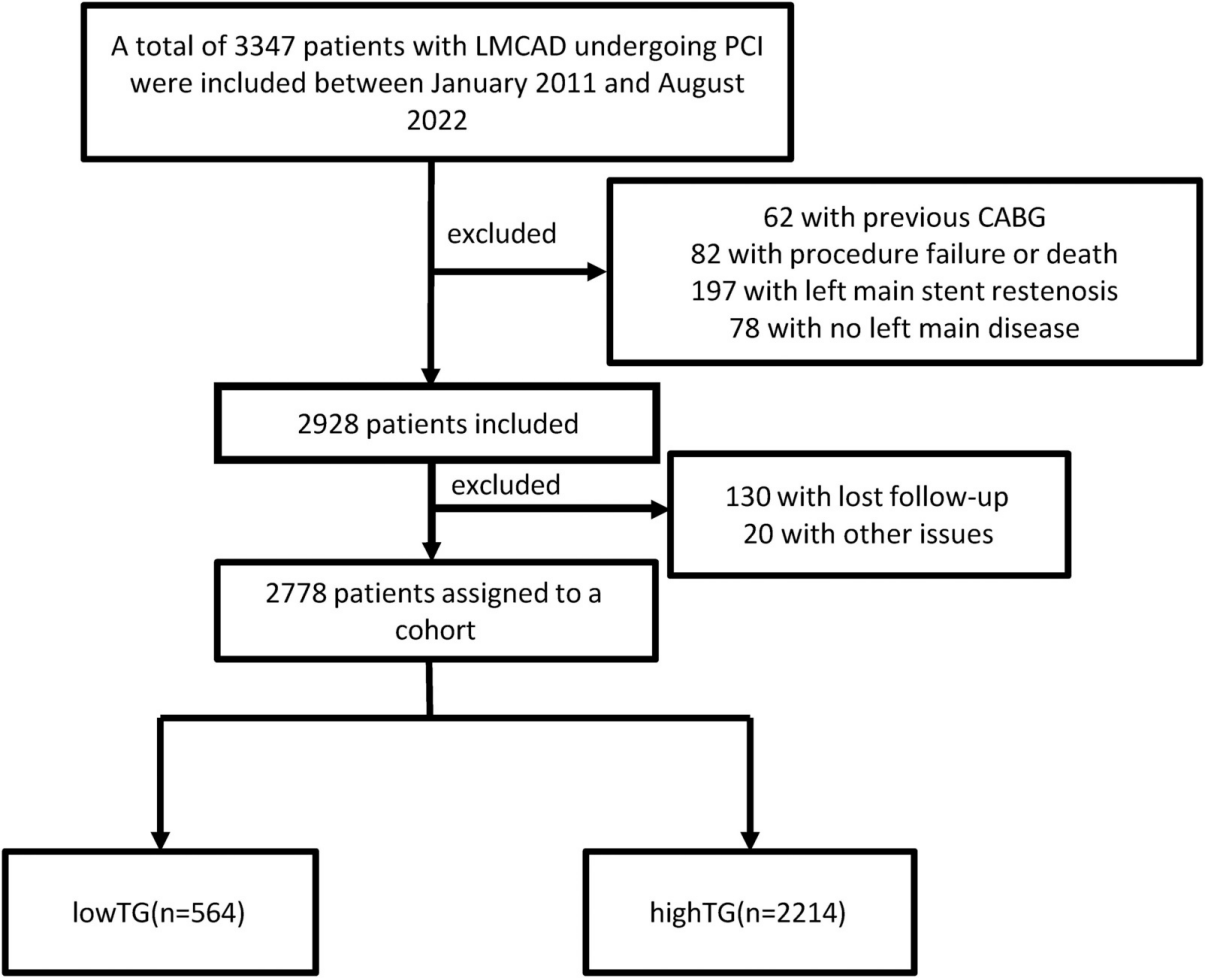
Flowchart of patient selection.

We identified 3,347 consecutive patients with LMCAD undergoing PCI at Nanjing First Hospital from January 2011 to August 2022. After excluding patients with prior CABG (62), procedural failure or death (82), in-stent restenosis (197), non-significant LM disease (78), lost follow-up (130), other reasons (20), including concurrent transcatheter aortic valve replacement (TAVR) during the PCI procedure (10), iatrogenic left main coronary artery dissection (7), and spontaneous left main coronary artery dissection (3), 2,778 patients remained for final analysis (Figure 1). PCI was performed according to current clinical guidelines. For the purpose of this study, PCI was defined as successful stent implantation in the left main coronary artery. Patients treated with percutaneous transluminal coronary angioplasty (PTCA) alone or those with failed stent delivery were excluded from the final analysis. Participants were then stratified by TG level. This study adhered to the Declaration of Helsinki and was approved by the institutional ethics committee (KY20170904-06); written consent was waived due to the retrospective design.

### 2.2 Data collection

All key procedural decisions were made by interventional cardiologists. Procedural success was defined as achieving Thrombolysis in Myocardial Infarction (TIMI) flow grade 3 with residual stenosis <30% (7). We collected data on clinical comorbidities (hypertension, dyslipidemia, and diabetes mellitus), medications, and laboratory values, including hemoglobin (Hb), low-density lipoprotein cholesterol (LDL-C), high-density lipoprotein (HDL-C) cholesterol, triglycerides (TG), fast blood glucose (FBG), creatine kinase MB (CK-MB), albumin, uric acid, and creatinine (Scr). We also recorded specific procedural data, including the number of LM stents, stent diameter and length, lesion location, intravascular ultrasound (IVUS), dual antiplatelet therapy (DAPT), and the use of intra-aortic balloon pump (IABP).

### 2.3 Clinical outcomes

All patients underwent regular follow-up via telephone interviews or clinical visits, with an initial minimum follow-up duration of 1 year. The primary endpoint was all-cause death. Secondary efficacy endpoints, defined according to the ULTIMATE-DAPT trial criteria (8), comprised cardiovascular death, myocardial infarction (MI), ischemic stroke, stent thrombosis (ST), and target vessel revascularization (TVR). The primary safety endpoint was major bleeding, defined as Bleeding Academic Research Consortium (BARC) types 3–5 (9).

### 2.4 Statistical analysis

Continuous variables are expressed as mean ± standard deviation (SD), and categorical variables are expressed as frequencies and percentages. Comparisons between groups were performed using Student's *t*-test or the Mann–Whitney *U* test for continuous data, and the Chi-square or Fisher's exact test for categorical data. The association between TG levels and mortality was modeled using restricted cubic splines (RCS) within a Cox proportional hazards framework. Nonlinearity was evaluated using likelihood ratio tests, and dose-response relationships were visualized using RCS curves with hazard ratios (HRs) and 95% confidence intervals (CIs) (10). The optimal TG cutoff for risk stratification was determined using maximally selected rank statistics (11). The proportional hazards assumption was verified using Schoenfeld residual plots, confirming its validity throughout the follow-up period. Survival outcomes across TG groups were compared using Kaplan–Meier curves and the log-rank test. Multivariable Cox proportional hazards analysis was performed using a block-wise entry approach. Established cardiovascular risk factors, including age, gender, BMI, LDL-C, and FBG, were force-entered (Block 1) into the model to ensure adequate adjustment. Other candidate laboratory and clinical variables were entered in the second block and subjected to a backward stepwise elimination process (Likelihood Ratio test) with a removal criterion of *P* > 0.05 (Block 2). This strategy ensured that the independent association between TG levels and mortality was adjusted for both statistically significant predictors and clinically essential confounders. Consistency of the TG-mortality association was evaluated through pre-specified subgroup analyses; interaction terms were included in the models, with *P* > 0.05 indicating a consistent effect across subgroups.

Statistical analyses were performed using IBM SPSS Statistics version 27.0 (IBM Corp., Armonk, NY, USA) and R software version 4.5.1 (R Foundation for Statistical Computing, Vienna, Austria). Specifically, baseline characteristics and Cox proportional hazards regression analyses were conducted in SPSS. R software, incorporating the rms and survival packages, was utilized for data visualization and advanced modeling, including the construction of the nomogram and Kaplan–Meier survival curves.

## 3 Results

### 3.1 Baseline characteristics

A total of 2,778 patients with LMCAD undergoing PCI were included in this study. The cohort's mean age was 67.34 ± 10.33 years, and 77.1% were male (Table 1). During a mean follow-up of 47.4 ± 30.3 months, 351 (12.6%) patients died, including 207 cardiovascular deaths.

**Table 1:** Baseline demographic, angiographic, and procedural characteristics between low and high TG groups.

Characteristics	Overall (*n* = 2778)	Low TG (*n* = 564)	High TG (*n* = 2214)	*P*-value
Age (y)	67.34 ± 10.33	70.10 ± 10.02	66.64 ± 10.29	<0.001
Male (*n*)	2143 (77.1)	490 (86.9)	1653 (74.7)	<0.001
BMI (kg/m2)	24.48 ± 3.16	23.40 ± 3.23	24.75 ± 3.08	<0.001
Heart rate (bpm)	73.67 ± 11.72	74.02 ± 12.36	73.58 ± 11.55	0.421
Mean blood pressure (mmHg)	96.31 ± 11.16	95.84 ± 11.15	96.43 ± 11.16	0.261
Hypertension (*n*)	1983 (71.4)	379 (67.2)	1604 (72.4)	0.014
DM (*n*)	931 (33.5)	175 (31.0)	756 (34.1)	0.161
Dyslipidemia (*n*)	2058 (74.1)	307 (54.4)	1751 (79.1)	<0.001
Prior myocardial infarction(*n*)	293 (10.5)	73 (12.9)	220 (9.9)	0.038
Prior PCI (*n*)	539 (19.4)	127 (22.5)	412 (18.6)	0.036
AMI (*n*)	651 (23.4)	128 (22.7)	523 (23.6)	0.643
Hb (g/L)	130.44 ± 17.28	126.45 ± 17.13	131.46 ± 17.18	<0.001
FBG (mmol/L)	6.23 ± 2.30	5.71 ± 1.95	6.36 ± 2.37	<0.001
Albumin (g/L)	38.25 ± 3.54	37.18 ± 3.86	38.52 ± 3.40	<0.001
Uric acid (umol/L)	344.33 ± 107.61	321.55 ± 97.11	350.13 ± 109.39	<0.001
LDL-C (mmol/L)	2.36 ± 1.47	1.95 ± 0.79	2.47 ± 1.58	<0.001
HDL-C (mmol/L)	0.98 ± 0.23	1.07 ± 0.26	0.96 ± 0.22	<0.001
Scr (umol/L)	86.21 ± 64.39	86.61 ± 55.61	86.11 ± 66.45	0.869
CK-MB (ng/mL)	18.05 ± 33.32	19.39 ± 40.37	17.71 ± 31.28	0.285
Ticagrelor (*n*)	1338 (48.2)	266 (47.2)	1072 (48.4)	0.594
ACE inhibitor or ARB (*n*)	1637 (58.9)	311 (55.1)	1326 (59.9)	0.041
Beta-blockers (*n*)	1780 (64.1)	325 (57.6)	1455 (65.7)	<0.001
Statins (*n*)	2716 (97.8)	548 (97.2)	2168 (97.9)	0.276
Oral Antidiabetic Drugs (*n*)	688 (24.8)	131 (23.2)	557 (25.2)	0.343
Insulin (*n*)	344 (12.4)	66 (11.7%)	278 (12.6)	0.582
DAPT (m)	15.33 ± 11.38	14.80 ± 10.08	15.46 ± 11.69	0.219
LM lesion location				0.856
Ostium (*n*)	246 (8.9)	47 (8.3)	199 (9.0)	
Shaft (*n*)	68 (2.4)	13 (2.3)	55 (2.5)	
Distal bifurcation (*n*)	2464 (88.7)	504 (89.4)	1960 (88.5)	
LM True-bifurcation (*n*)	1323 (47.6)	265 (47.0)	1058 (47.8)	0.734
triple-vessel disease (*n*)	1441 (51.9)	297 (52.7)	1144 (51.7)	0.675
Length of LM stent (mm)	25.29 ± 7.62	25.17 ± 7.18	25.32 ± 7.73	0.675
Diameter of LM stent (mm)	3.43 ± 0.35	3.42 ± 0.35	3.43 ± 0.35	0.596
2-stent strategy (*n*)	679 (24.4)	139 (24.6)	540 (24.4)	0.900
IVUS (*n*)	1046 (37.7)	212 (37.6)	834 (37.7)	0.972
IABP (*n*)	175 (6.3)	35 (6.2)	140 (6.3)	0.918

Values are *n* (%) or mean ± SD. BMI, body mass index; DM, diabetes; PCI, percutaneous coronary intervention; AMI, acute myocardial infarction; Hb, hemoglobin; LDL-C, low-density lipoprotein cholesterol; HDL-C, high-density lipoprotein cholesterol; TG, triglyceride; FBG, fasting blood glucose; Scr, serum creatinine; CK-MB, creatine kinase MB; DAPT, dual antiplatelet therapy; ARB, angiotensin receptor blocker; ACEI, angiotensin-converting enzyme inhibitor. IVUS, intravascular ultrasound; LM, left main; IABP, intra-aortic balloon pump. True bifurcation was defined as a Medina classification type of 1,1,1, 1,0,1, or 0,1,1.

### 3.2 Univariate and multivariable predictors of all-cause mortality

Univariate analysis screened 23 potential variables associated with all-cause mortality (Table 2). Subsequent multivariable Cox regression analysis confirmed that age, diabetes mellitus, prior MI, hemoglobin, albumin, uric acid, FBG, TG, creatinine, CK-MB, DAPT duration, triple-vessel disease, LM stent diameter, and IABP use remained independent predictors of all-cause mortality.

**Table 2:** Univariate and multivariable Cox proportional hazards analysis of predictors for all-cause mortality in LMCAD patients undergoing percutaneous coronary intervention.

Covariates	HR (95%CI)	*P*-value	HR (95%CI)	*P*-value
Age, per 5 years increase	1.36 (1.28–1.445)	<0.001	1.259 (1.179–1.344)	<0.001
Male	0.958 (0.748–1.226)	0.732	0.909 (0.691–1.195)	0.492
BMI (kg/m2)	0.942 (0.91–0.975)	0.001	1.000 (0.966–1.036)	0.983
Heart rate, per 5 bpm increase	1.095 (1.053–1.138)	<0.001		
Mean blood pressure, per 5 mmHg increase	0.998 (0.952–1.047)	0.937		
Hypertension	1.411 (1.093–1.822)	0.008		
DM	1.577 (1.276–1.948)	<0.001	1.338 (1.045–1.715)	0.021
Dyslipidemia	1.119 (0.871–1.439)	0.378		
Prior MI	1.802 (1.357–2.393)	<0.001	1.740 (1.298–2.332)	<0.001
Prior PCI	1.302 (1.019–1.664)	0.035		
AMI	1.576 (1.256–1.977)	<0.001		
Hb, per 5 g/L increase	0.860 (0.835–0.886)	<0.001	0.927 (0.895–0.960)	<0.001
Albumin (g/L)	0.873 (0.848–0.899)	<0.001	0.941 (0.912–0.972)	<0.001
Uric acid, per 50 umol/L increase	1.152 (1.104–1.203)	<0.001	1.108 (1.060–1.157)	<0.001
LDL-C (mmol/L)	0.953 (0.854–1.064)	0.391	1.026 (0.966–1.090)	0.406
FBG (mmol/L)	1.077 (1.039–1.117)	<0.001	1.068 (1.023–1.114)	0.003
TG (mmol/L)	0.730 (0.624–0.855)	<0.001	0.815 (0.688–0.966)	0.018
HDL-C (mmol/L)	0.578 (0.355–0.942)	0.028		
Scr, per 5 umol/L increase	1.013 (1.010–1.016)	<0.001	1.008 (1.004–1.012)	<0.001
CK-MB, per 20 ng/mL	1.046 (1.009–1.084)	0.014	1.073 (1.029–1.119)	0.001
Ticagrelor	0.730 (0.630–1.007)	0.057		
ACE inhibitor or ARB	0.873 (0.706–1.081)	0.214		
Betablockers	0.930 (0.748–1.156)	0.514		
Statins	1.552 (0.642–3.752)	0.330		
Oral Antidiabetic Drugs	1.223 (0.967–1.547)	0.092		
Insulin	2.049 (1.574–2.668)	<0.001		
DAPT (m)	0.979 (0.967–0.991)	0.001	0.967 (0.955–0.980)	<0.001
LM distal bifurcation	1.329 (0.937–1.885)	0.111		
LM True-bifurcation	1.495 (1.211–1.846)	<0.001		
Triple-vessel disease	1.892 (1.519–2.356)	<0.001	1.405 (1.119–1.765)	0.003
Length of LM stent	1.000 (0.986–1.014)	0.967		
Diameter of LM stent	0.442 (0.327–0.597)	<0.001	0.597 (0.434–0.820)	0.001
2-stent strategy	1.327 (1.060–1.660)	0.013		
IVUS	0.824 (0.661–1.027)	0.085		
IABP	2.634 (1.952–3.553)	<0.001	1.455 (1.059–1.999)	0.021

Values are *n* (%) or mean ± SD. BMI, body mass index; DM, diabetes; PCI, percutaneous coronary intervention; AMI, acute myocardial infarction; Hb, hemoglobin; LDL-C, low-density lipoprotein cholesterol; HDL-C, high-density lipoprotein cholesterol; TG, triglyceride; FBG, fasting blood glucose; Scr, serum creatinine; CK-MB, creatine kinase MB; DAPT, dual antiplatelet therapy; ARB, angiotensin receptor blocker; ACEI, angiotensin-converting enzyme inhibitor. IVUS, intravascular ultrasound; LM, left main; IABP, intra-aortic balloon pump.

### 3.3 Dose-response relationship between triglyceride levels and mortality risk

**Figure 2:**
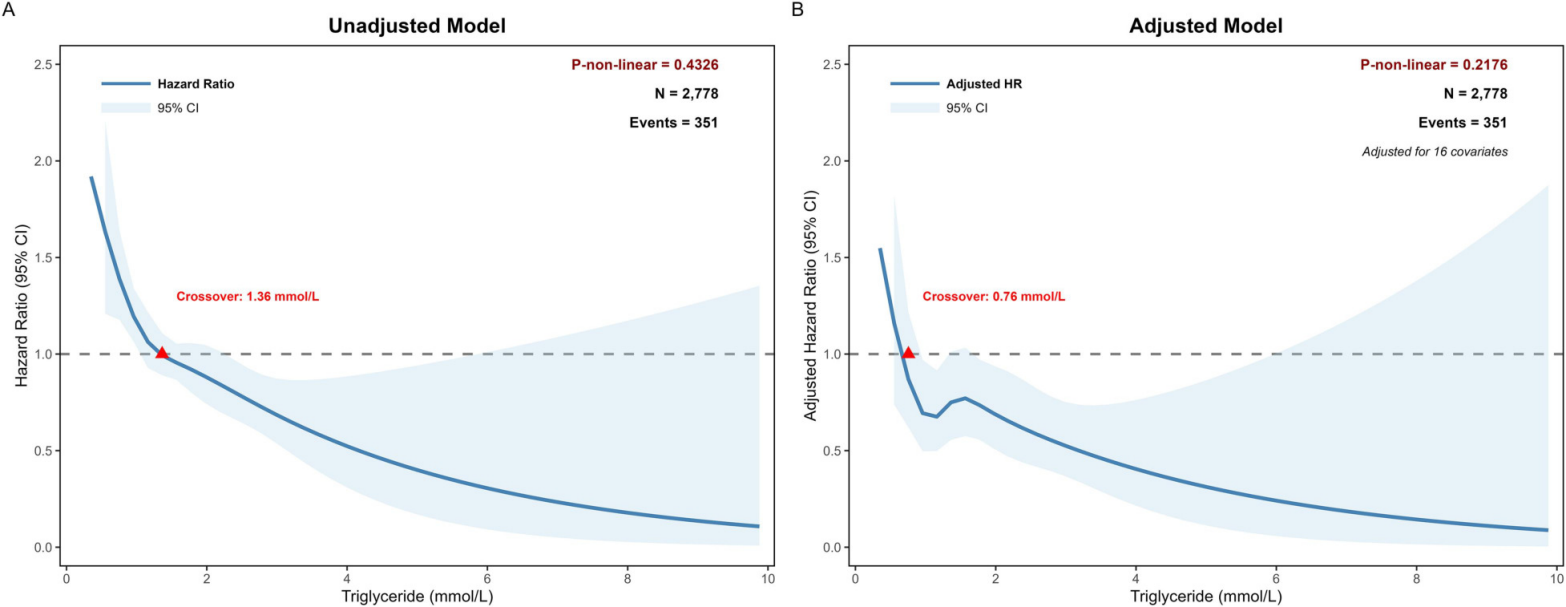
Restricted cubic spline (RCS) curves for the association between triglyceride levels and all-cause mortality. **(A)** Unadjusted Cox proportional hazards model demonstrating the hazard ratio (HR) for TG levels. **(B)** Multivariable-adjusted model, accounting for Age, gender, BMI, LDL-C, FBG, DM, Prior MI, Hemoglobin, Albumin, Uric acid, creatinine, CK-MB, DAPT, Triple-vessel disease, LM stent Diameter, and IABP. The solid lines represent the HRs, and the shaded areas indicate the 95% confidence intervals. The likelihood ratio test confirmed a persistent, non-linear relationship that did not reach statistical significance after adjustment (*P* for nonlinearity = 0.2176).

Both univariate and multivariable RCS analyses revealed a significant linear inverse association between TG levels and all-cause mortality (P-non-linea*r* = 0.4326 and 0.2176, respectively). The multivariable model was rigorously adjusted for age, gender, BMI, LDL-C, FBG, DM, prior MI, hemoglobin, albumin, uric acid, creatinine, CK-MB, DAPT, triple-vessel disease, LM stent diameter, and IABP. In the crude model, the hazard ratio (HR) crossed the point of neutrality at a TG threshold of 1.36 mmol/L; notably, this threshold shifted to 0.76 mmol/L after adjusting for the aforementioned clinical and laboratory confounders (Figure 2).

### 3.4 Risk stratification using maximally selected rank statistics

**Figure 3:**
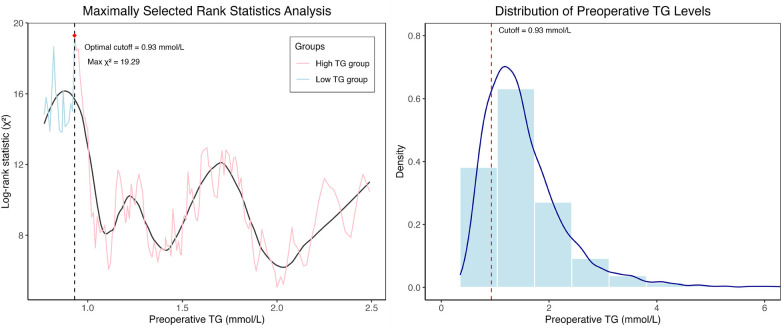
Distribution of TG levels and the identified optimal cutoff value.

**Figure 4:**
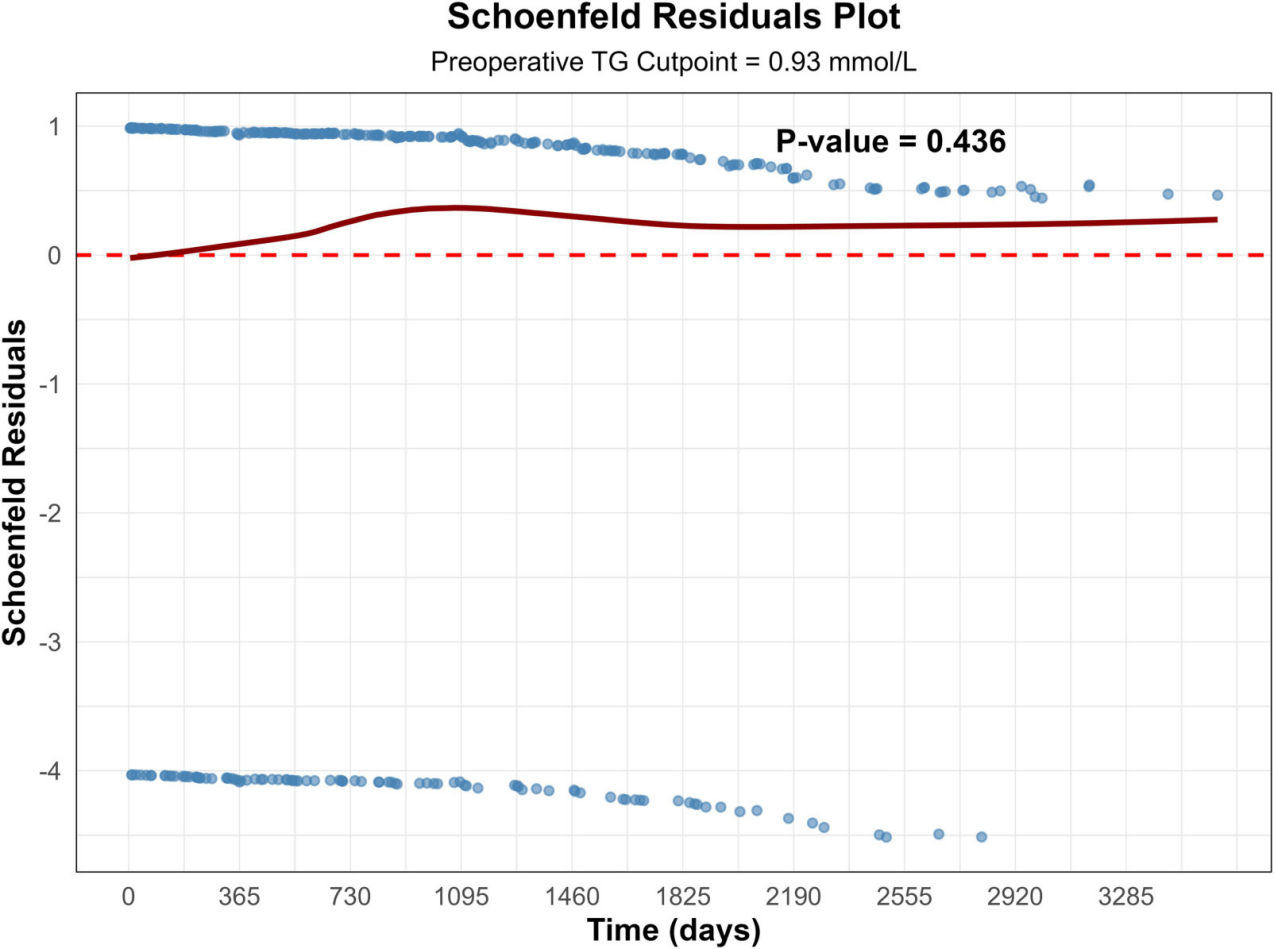
Schoenfeld residual plots for proportional hazards assumption.

We performed maximally selected rank statistics to identify the optimal TG cutoff for risk stratification of all-cause mortality. Using the maximally selected rank statistic approach, an optimal TG cutoff of 0.93 mmol/L was identified for risk stratification, corresponding to a maximum log-rank statistic of 19.29 (Figure 3). Patients were then categorized into a high-TG group (≥0.93 mmol/L, *n* = 2214) and a low-TG group (<0.93 mmol/L, *n* = 564). Evaluation of the proportional hazards assumption using Schoenfeld residuals confirmed the validity of the Cox model (global test *P* = 0.436), indicating that the effect of TG stratification remained consistent throughout follow-up (Figure 4).

### 3.5 Endpoint analysis

**Figure 5:**
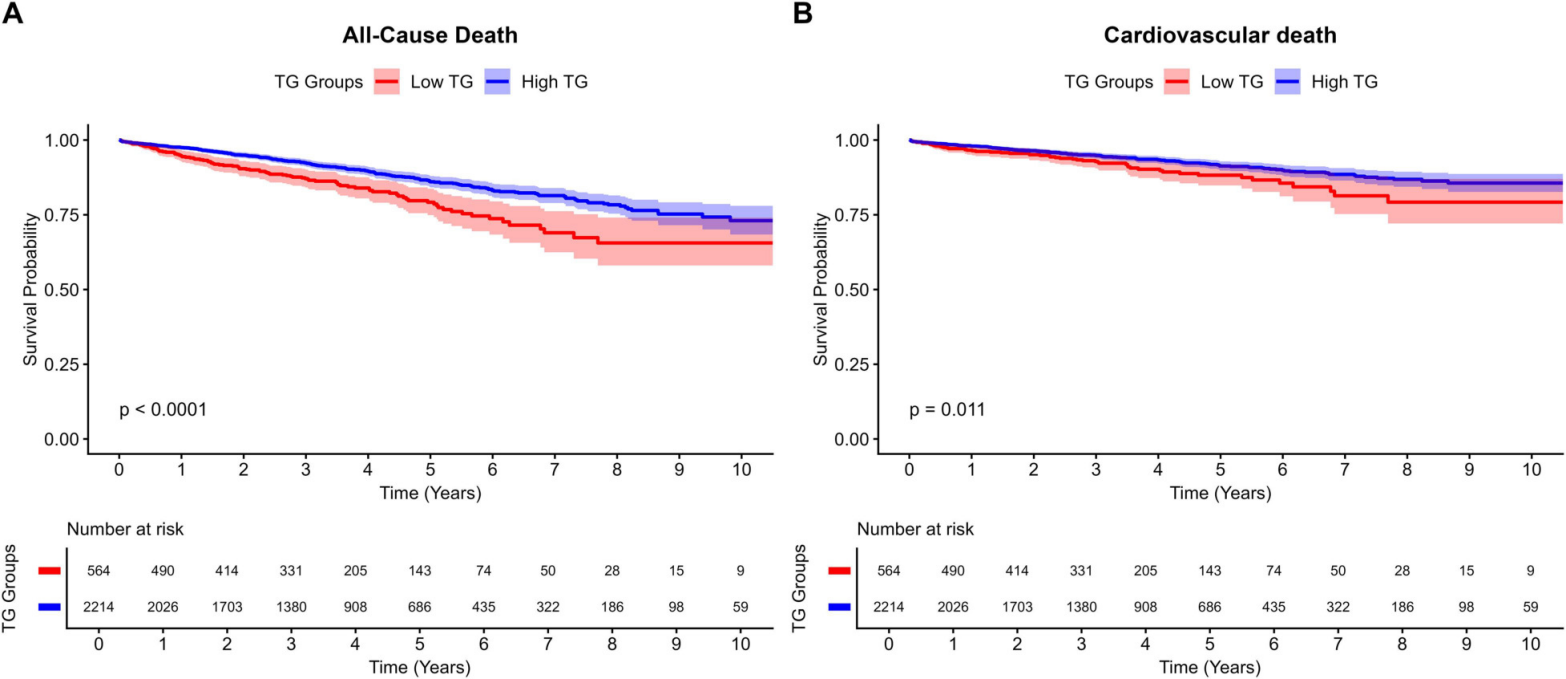
Kaplan-Meier curves for **(A)** all-cause death and **(B)** cardiovascular death between TG groups.

Compared with the high-TG group, patients in the low-TG cohort were older and exhibited significantly lower body mass index (BMI) and lower levels of hemoglobin, albumin, LDL-C, FBG, and uric acid (Table 1). Kaplan–Meier analysis (Figure 5A) showed a significantly lower cumulative survival rate in the low-TG group than in the high-TG group (*P* < 0.001, log-rank test). After multivariable adjustment, low TG levels remained independently associated with an increased risk of all-cause mortality (17.2% vs. 11.5%; adjusted HR: 1.379; 95% CI: 1.069–1.778; *P* = 0.013; Table 3). Similarly, the low-TG group experienced higher cardiovascular mortality (Log-rank *P* = 0.011; Figure 5B), which persisted as an independent predictor after adjusting for potential confounders (adjusted HR: 1.458; 95% CI: 1.041–2.043; *P* = 0.028). Notably, no significant differences were observed in other secondary endpoints, including myocardial infarction, stroke, target vessel revascularization, stent thrombosis, or major bleeding (BARC 3–5).

**Table 3:** Clinical outcomes between low and high TG groups.

Clinical outcomes	Low TG	High TG	Univariable HR (95%CI)	*P*-value	Multivariable HR (95%CI)	*P*-value
Primary efficacy endpoint						
All-cause death	97 (17.2%)	254 (11.5%)	1.681 (1.330–2.125)	˂0.001	1.379 (1.069–1.778)	0.013
Secondary efficacy endpoint						
Cardiovascular death	53 (9.4%)	154 (7.0%)	1.493 (1.093–2.042)	0.012	1.458 (1.041–2.043)	0.028
Myocardial infarction	17 (3.0%)	75 (3.4%)	0.976 (0.576–1.655)	0.928	0.959 (0.547–1.681)	0.884
Stroke	15 (2.7%)	36 (1.6%)	1.767 (0.967–3.229)	0.064	1.758 (0.928–3.332)	0.084
Target vessel revascularization	61 (10.8%)	235 (10.6%)	1.110 (0.837–1.472)	0.468	1.209 (0.895–1.633)	0.215
Stent thrombosis	33 (5.9%)	104 (4.7%)	1.362 (0.920–2.015)	0.123	1.381 (0.908–2.100)	0.131
Primary safety endpoint						
Major bleeding (BARC3, 5)	11 (2.0%)	48 (2.2%)	0.950 (0.493–1.829)	0.877	0.787 (0.396–1.563)	0.494

Values are presented as *n* (%) or mean ± SD. HR, hazard ratio; CI, confidence interval; TG, triglyceride; ST, stent thrombosis; BARC, Bleeding Academic Research Consortium. All models were adjusted for a comprehensive set of covariates, including age, sex, BMI, LDL-C, FBG, diabetes mellitus, prior MI, hemoglobin, albumin, uric acid, creatinine, CK-MB, DAPT, triple-vessel disease, LM stent diameter, and IABP. For stroke and bleeding endpoints, a parsimonious multivariable model incorporating core clinical risk factors (age, sex, BMI, LDL-C, and FBG) was utilized due to the limited number of events.

Major adverse cardiovascular events (MACE) were defined as a composite of cardiovascular death, non-fatal myocardial infarction, and non-fatal stroke. Kaplan–Meier analysis revealed that the low TG group had a significantly higher incidence of MACE compared to the higher TG group (log-rank *P* = 0.035). However, this association was attenuated after adjusting for potential confounders in the multivariable Cox proportional hazards model (adjusted hazard ratio 1.285; 95% CI: 0.970–1.703; *P* = 0.081) (Supplementary Table S1). The discrepancy between MACE and cardiovascular death suggests that the prognostic value of TG in this cohort is primarily driven by fatal outcomes rather than non-fatal ischemic events.

### 3.6 Subgroup analyses

After adjusting for the same set of covariates as in the main model, subgroup analyses showed a consistently higher mortality risk in the low-TG group across all predefined strata. No significant interactions were observed between TG levels and the subgroup variables (all P-interactions >0.05), suggesting that the prognostic value of TG stratification remains robust across baseline clinical characteristics. Detailed results are provided in Table 4.

**Table 4:** Subgroup analysis of the association between TG levels and all-cause mortality.

Covariates	Events	HR (95%CI)	*P*-value	Interaction *P*-value
Total	351/2778	1.370 (1.062–1.768)	0.015	
Age <75	183/2033	1.229 (0.838–1.803)	0.291	0.687
Age ≥75	168/745	1.448 (1.018–2.060)	0.039	
No-triple-vessel disease	124/1337	1.224 (0.780–1.923)	0.380	0.617
Triple-vessel disease	227/1441	1.555 (1.134–2.131)	0.006	
No-AMI	243/2127	1.240 (0.908–1.693)	0.176	0.395
AMI	108/651	1.575 (0.979–2.533)	0.061	
No-DM	202/1847	1.499 (1.079–2.084)	0.016	0.767
DM	149/931	1.248 (0.825–1.890)	0.294	
Female	82/635	1.689 (0.898–3.178)	0.104	0.319
Male	269/2143	1.330 (1.003–1.763)	0.047	
Ticagrelor	108/1338	1.048 (0.652–1.686)	0.845	0.207
Clopidogrel	243/1440	1.568 (1.152–2.133)	0.004	

Values are *n* (%) or mean ± SD. HR, hazard ratio; UA, unstable angina; AMI, acute myocardial infarction; DM, diabetes.

## 4 Discussion

Our findings demonstrate that after rigorous adjustment for cardiovascular risk factors, elevated fasting serum TG levels are independently associated with a reduced risk of all-cause mortality in patients with LMCAD undergoing PCI. The consistency of this association across all predefined subgroups further underscores the robustness of our results. Nevertheless, the prognostic significance of serum TG levels in cardiovascular disease (CVD) remains a subject of intense debate (12). This lack of consensus in the literature may be attributed to heterogeneity in study populations, variable control of confounding factors, and diverse therapeutic strategies across cohorts (13).

Toth et al. (14) utilized propensity score matching in patients with diabetes or ASCVD, demonstrating that elevated triglycerides (TG: 2.26–5.64 mmol/L) were associated with a significantly higher risk of composite cardiovascular endpoints and increased healthcare costs compared to the low-TG group. Similarly, Nelson et al. (15) reported that participants in the BARI 2D trial with TG ≥ 150 mg/dL had a higher risk profile, characterized by younger age, higher BMI, lower HDL-C, and elevated HbA1c. Beyond these clinical observations, Mendelian randomization by Holmes et al. (16) and a meta-analysis by Assempoor et al. (17) have further corroborated a causal link between elevated TG and adverse ASCVD outcomes. These studies collectively reinforce the traditional view of TG as a primary driver of cardiovascular risk.

Contrary to the conventional view that elevated TG is universally deleterious, emerging evidence suggests that low TG levels may be paradoxically associated with adverse cardiovascular outcomes. For instance, in a cohort of 3,061 patients with angiographically confirmed coronary artery disease, Xia et al. (18) stratified participants into three TG tiers (<1.18, 1.18–1.82, >1.82 mmol/L). Their multivariable Cox analysis revealed that the highest TG group had a significantly lower risk of all-cause mortality than the lowest TG group. Similarly, Cheng et al. (19) documented an inverse relationship between elevated TG levels and the incidence of target vessel revascularization (TVR) and major adverse cardiovascular events (MACE) in STEMI patients undergoing primary PCI.

However, the relationship between TG levels and clinical outcomes appears increasingly complex in specific patient populations. Ren et al. (10) observed that among patients with heart failure, the association was not monotonic; their adjusted models showed that both suboptimal (<1.2 mmol/L) and excessive (>3.0 mmol/L) TG levels were significantly associated with higher mortality. Similarly, in an analysis of National Health and Nutrition Examination Survey (NHANES) data, Huang et al. (20) identified a distinct U-shaped relationship between TG levels and all-cause mortality. Their restricted cubic spline (RCS) or smoothing spline plots indicated that the nadir of risk (lowest HR) occurred at a TG concentration of approximately 135 mg/dL, with mortality risk rising at either extreme of the lipid spectrum.

It is essential to recognize that serum TG levels are primarily determined by the metabolism of triglyceride-rich lipoproteins (TGRLs). While debate persists regarding whether TG is a direct causal agent or merely a surrogate marker of cardiovascular risk, substantial evidence indicates that TGRLs—specifically chylomicrons and very-low-density lipoproteins (VLDL)—are directly implicated in the pathogenesis of ASCVD (21). Beyond atherosclerosis, circulating TG levels often reflect a patient's broader nutritional status and adipose tissue distribution. This is corroborated by the World Health Organization Stepwise Approach to Surveillance (WHO STEPS) data (2000–2020), which demonstrated a robust positive association between TG levels and abdominal obesity (22). We propose that low TG may not merely be a laboratory finding but also a sign of poorer overall organ health and reduced capacity to handle physiological stress.

Altered tumor metabolism, which modulates systemic TG levels, is well-exemplified by the inhibition of ceramide synthase 5 (CERS5). In a murine hepatocellular carcinoma model, CERS5 suppression not only hindered tumor progression but also significantly reduced TG accumulation (23). Clinical insights from the INSCOC trial by Tian et al. (24) further highlight the nuanced role of TG in cancer, demonstrating that its prognostic value depends on body composition. Specifically, low TG levels emerged as an independent risk factor for poor prognosis in cachectic patients, yet functioned as a protective factor in obese individuals. Moreover, in patients undergoing immunotherapy, low TG levels were linked to a higher incidence of immune-related adverse events, suggesting a fundamental intersection between lipid metabolism and anti-tumor immunity (25).

Beyond oncology, the sympathetic nervous system, acutely activated during coronary events, can upregulate lipolytic genes, thereby accelerating TG breakdown (26). This is consistent with an observational study by Cheng et al. (27), which found that TG levels were significantly lower in patients with acute myocardial infarction (AMI). This hypolipidemic state was independently associated with a heightened risk of recurrent ischemia and increased 30-day in-hospital mortality. We propose that TG synthesis serves as a critical protective mechanism against lipotoxicity by sequestering excess free fatty acids (28). This physiological role mirrors that of B-type natriuretic peptide (BNP), which rises as a compensatory response to cardiac wall stress. Consequently, circulating TG levels may function as a systemic biomarker, reflecting both the severity of the primary injury and the subsequent prognosis in AMI patients (29).

The present study demonstrates that low serum TG levels are independent predictors of both all-cause and cardiovascular mortality in patients with LM diseases undergoing PCI. Specifically, our findings reveal a “lipid paradox” where lower TG concentrations-traditionally viewed as a marker of lower cardiovascular risk-are paradoxically associated with a significantly higher risk of fatal outcomes. Several potential mechanisms may explain this association. First, low TG levels often serve as a surrogate marker for malnutrition and a frailty state, conditions frequently observed in elderly or chronically ill cardiac patients (27). This “malnutrition-inflammation complex” may lead to reduced physiological reserves and impaired recovery following PCI. Second, extremely low TG concentrations may reflect a state of chronic systemic inflammation, which is known to downregulate lipid metabolism while simultaneously accelerating atherosclerosis and myocardial damage (30). Interestingly, while low TG was strongly predictive of mortality, its association with non-fatal ischemic events (such as MACE) was less robust. This suggests that the prognostic value of low TG in this population is primarily driven by an increased susceptibility to fatal complications rather than a direct promotion of non-fatal thrombotic episodes. These results underscore the importance of monitoring lipid profiles not only for hyperlipidemia but also as a holistic indicator of a patient's nutritional and systemic health status.

The clinical significance of low TG levels is especially noteworthy within the context of left main disease. Unlike patients with single-vessel or peripheral coronary lesions, those with LM disease possess a significantly larger area of “myocardium at risk,” where any hemodynamic instability or systemic complication can be life-threatening. In this high-stakes cohort, low TG serves as more than just a metabolic marker; it reflects a state of diminished “biological buffering capacity.” Patients with low TG, often presenting with subclinical malnutrition or a frailty phenotype, may lack the physiological resilience required to withstand the significant systemic stress associated with complex PCI and the subsequent long-term recovery process (31). Our findings suggest that while low TG does not necessarily increase the risk of focal mechanical failures (such as stent thrombosis), it identifies a subset of “fragile” patients who are at a disproportionately higher risk of fatal cardiovascular collapse. Therefore, in the management of LM disease, integrating nutritional and metabolic assessment via TG levels may provide a more holistic risk stratification beyond traditional anatomical scoring systems.

Several limitations must be considered when interpreting our findings. First, the retrospective, single-center observational design is inherently susceptible to selection bias and unmeasured confounding, despite our rigorous multivariate adjustments. Second, the modest sample size may have constrained our statistical power to detect more subtle associations. Third, data on left ventricular systolic function (e.g., left ventricular ejection fraction and B-type natriuretic peptide) and the anatomical complexity of coronary lesions (e.g., SYNTAX score) were unavailable for this cohort. Given that these factors are established prognostic determinants, their absence may introduce unmeasured confounding into our survival analysis. Finally, our analysis relied on a single preoperative serum TG measurement, which does not account for longitudinal fluctuations or dynamic changes over the follow-up period.

## 5 Conclusion

Our study identified a paradoxical inverse association between low TG levels and all-cause mortality in patients with LMCAD undergoing PCI. These findings challenge the conventional paradigm that elevated lipid levels are universally detrimental and underscore the potential existence of protective metabolic mechanisms inherent to TG within this high-risk CAD phenotype.

## Data Availability

The raw data supporting the conclusions of this article will be made available by the authors, without undue reservation.

## Supplementary material

Supplementary Table S1 MACE between low and high TG groups.
