# Supplementary material for: Triglyceride levels and all-cause mortality in patients with left main coronary artery disease undergoing percutaneous coronary intervention
Source: Front Cardiovasc Med. 2026 Apr 23;13:1751611. doi: 10.3389/fcvm.2026.1751611 (PMC13149153; doi:10.3389/fcvm.2026.1751611)
Supplement: Supplementary Table S1 — MACE between low and high TG groups. [file Table1.docx]

**Supplementary Table 1: MACE between Low and high TG groups**

|  | **Low TG** | **High TG** | **Univariable HR (95%CI)** | **P-value** | **Multivariable HR (95%CI)** | **P-value** |
| --- | --- | --- | --- | --- | --- | --- |
| **MACE** | 73 (12.9%) | 236 (10.7%) | 1.326 (1.019-1.724) | 0.036 | 1.285 (0.970-1.703) | 0.081 |
| Cardiovascular death | 53 (9.4%) | 154 (7.0%) | 1.493 (1.093-2.042) | 0.012 | 1.458 (1.041-2.043) | 0.028 |
| Non-fatal Myocardial Infarction | 11 (2.0%) | 64 (2.9%) | 0.742 (0.391-1.408) | 0.361 | 0.732 (0.373-1.436) | 0.364 |
| Non-fatal Stroke | 11 (2.0%) | 32 (1.4%) | 1.441 (0.726-2.860) | 0.296 | 1.364 (0.665-2.798) | 0.397 |

Values are presented as n (%) or mean ± SD. HR, hazard ratio; CI, confidence interval; MACE, major adverse cardiovascular events. All models were adjusted for a comprehensive set of covariates, including age, sex, BMI, LDL-C, FBG, diabetes mellitus, prior MI, hemoglobin, albumin, uric acid, creatinine, CK-MB, DAPT, triple-vessel disease, LM stent diameter, and IABP. For non-fatal stroke endpoints, a parsimonious multivariable model incorporating core clinical risk factors (age, sex, BMI, LDL-C, and FBG) was utilized due to the limited number of events.
